# Quinine-Resistant Malaria in Traveler Returning from French Guiana, 2010

**DOI:** 10.3201/eid1705.101424

**Published:** 2011-05

**Authors:** Lionel Bertaux, Philippe Kraemer, Nicolas Taudon, Aurélie Trignol, Maryse Martelloni, Redouan Saidi, Daniel Parzy, Bruno Pradines, Fabrice Simon

**Affiliations:** Author affiliations: Institut de Recherche Biomédicale des Armées, Marseille, France (L. Bertaux, N. Taudon, M. Martelloni, D. Parzy, B. Pradines);; Laveran Military Teaching Hospital, Marseille (P. Kraemer, A. Trignol, R. Saidi, F. Simon)

**Keywords:** parasites, malaria, quinine resistance, plasmodium falciparum, antimalarial drug resistance, French Guiana, antimicrobial resistance, letter

**To the Editor:** Resistance of *Plasmodium falciparum* to antimalarial drugs is one of the most worrying problems in tropical medicine. For *P. falciparum* malaria acquired in French Guiana, the combination of quinine and doxycycline is one of the first-line recommended treatments (*1*). Since 1996, only 2 treatment failures with quinine have been reported from that country (*2*). An elevated 50% inhibitory concentration (IC_50_), classified as in vitro quinine resistance, was reported for 17% of 32 *P. falciparum* isolates obtained during 1983–1987 in French Guiana (*3*). Throughout 1994–2005, isolates were susceptible to quinine, with a mean IC_50_ <200 nmol/L (*4*).

We report quinine treatment failure in a 35-year-old man who was infected during a 3-month stay in Saül, a rural area of French Guiana. The patient did not use antivectorial or antimalarial prophylaxis. The patient sought treatment with fever 4 days after returning to France on June 22, 2010 (day 0), and a diagnosis of *P. falciparum* malaria was made on the basis of results of a rapid diagnostic test performed by a private medical laboratory. The man, who weighed 58 kg, was treated as an outpatient with 500 mg of quinine to be taken orally 3×/d for 7 days; he did not receive doxycycline. He was admitted to the Laveran Military Teaching Hospital in Marseille on July 15 (day 24 and first day of recrudescence) for uncomplicated malaria with a *P. falciparum* parasitemia level of 4%. He was given artemether, 80 mg/d, by intramuscular injection for 3 days. Blood samples taken on day 27 (third day of recrudescence) and day 52 (4 weeks of recrudescence) were negative for *P. falciparum*.

In vitro testing of drug susceptibility was performed by the standard 42-hour ^3^H-hypoxanthine uptake inhibition method (*5*). We assessed susceptibility to 11 antimalarial drugs on the fresh isolate and after culture adaptation (Table). The laboratory-adapted strain 3D7, tested 3× on the same batch of plates, was used as reference. The strain isolated from the blood sample on day 24 (first day of the recrudescence) showed reduced susceptibility to quinine (1,019 nmol/L), chloroquine (427 nmol/L), and monodesethylamodiaquine (157 nmol/L). The isolate was susceptible to all other antimalarial drugs tested. We assessed gene polymorphisms of *pfcrt* (*P. falciparum* chloroquine resistance transporter), *pfmdr1* (*P. falciparum* multidrug resistance 1 protein), and *pfnhe-1* (*P. falciparum* Na^+^/H^+^ exporter 1); the copy number of *pfmdr1* involved in quinoline resistance; and gene polymorphisms of *dhfr* (dihydrofolate reductase, involved in proguanil or pyrimethamine resistance), *dhps* (dihydropteroate synthetase, involved in sulfadoxine resistance), and *cytB* (cytochrome B, involved in atovaquone resistance) (*6*).

**Table:** In vitro susceptibility to standard antimalarial drugs of a fresh isolate of *Plasmodium falciparum* and after culture adaptation in comparison with *P. falciparum* 3D7 clone tested with the same plate batches*

Antimalarial drug	Fresh isolate IC_50_, nmol/L	Cultured isolate IC_50_, nmol/L, mean ± SD†	3D7 clone IC_50_, nmol/L, mean ± SD	IC_50_, isolate:3D7	Resistance cutoff value, nmol/L
Quinine	1,019	1,087 ± 145	171 ± 26	6.0:6.4	>800
Chloroquine	427	492 ± 39	13.9 ± 2.2	30.7:35.4	>100
Monodesethylamodiaquine	157	157 ± 27	16.3 ± 3.5	9.6:9.6	>80
Mefloquine	16.4	14.9 ± 2.2	53.4 ± 5.2	0.31:0.28	>30
Lumefantrine	8.9	9.0 ± 3.0	46.0 ± 6.0	0.19:0.20	>150
Pyronaridine	45.2	37.8 ± 8.4	18.3 ± 1.6	2.5:2.1	ND
Piperaquine	80.4	99.5 ± 11.7	56.3 ± 5.4	1.4:1.8	ND
Dihydroartemisinin	2.18	2.46 ± 0.28	2.58 ± 0.13	0.84:0.95	>10.5
Artesunate	1.86	1.68 ± 0.28	2.27 ± 1.15	0.82:0.74	>10.5
Atovaquone	6.5	5.2 ± 0.8	4.2 ± 0.7	1.5:1.2	>490
Doxycycline	11,600	12,600 ± 1,700	12,600 ± 1,000	0.92:1.0	>35,000

*Isolate was obtained from a man who returned to France from French Guiana in June 2010. IC50, 50% inhibitory concentration; ND, not determined. †Mean IC50 and standard deviation of 3 different tests on the isolate after culture adaptation.

The *pfnhe* ms4760 microsatellite showed a profile 3, with 1 repeat of DNNND and 2 repeats of DDDNHNDNHNN. Studies of the *pfnhe-1* polymorphism of worldwide culture-adapted isolates showed that increased numbers of DNNND were associated with decreased quinine susceptibility (*7*). Association of 2 repeats of DNNND and a high quinine IC_50_ value was found in a case of clinical failure of quinine in a traveler returning from Senegal (*8*).

Reinfection was excluded because the patient had stayed in mainland France since his return. The patient reported that he took the quinine as instructed. We report here a clinical and parasitologic failure of quinine treatment associated with high IC_50_ but not linked with the ms4760 *pfnhe-1* profile involved with quinine in vitro reduced susceptibility. A hypothesis that may explain our data is that unlike previous studies (*7*), in which the less susceptible strains originated from Asia, this isolate came from South America. The profile associated with quinine resistance for chloroquine resistance and *pfcrt* genotypes could be different in the 3 malaria-endemic continents. There are no data on ms4760 *pfnhe-1* of *P. falciparum* isolates from South America. In another recent study (*9*), a multivariate analysis performed on 83 clinical isolates from Madagascar and 13 African countries did not confirm this association between quinine susceptibility and *pfnhe-1* microsatellite polymorphisms.

The *pfcrt* gene had a point mutation on codon 76 (76T) and *pfmdr1* on codons 184F, 1034C, 1042D, and 1246Y. These data are in agreement with those from previous studies that showed the mutation 76T in the *pfcrt* gene led to decreased susceptibility to chloroquine, amodiaquine, and quinine. The isolate had only 1 copy of *pfmdr1*. The data on mutations and copy number of *pfmdr1* are consistent with data in Brazil (*10*). Nevertheless, the lack of gene amplification and specific point mutations in *pfmdr1* were not associated with decreased in vitro susceptibility of quinine. *dhfr* and *dhps* genes had a 5-mutation haplotype, 51I C59 108N I164-S436 437G 540E 581G A613, which suggested in vitro resistance to proguanil, pyrimethamine, and sulfadoxine. This case confirms the need to always add doxycycline to quinine for treatment of *P. falciparum* malaria acquired in French Guiana as well as other parts of South America.
